# A Study on the Influencing Factors of the Mechanical Properties of Steel-Fiber-Reinforced Cement Concrete

**DOI:** 10.3390/ma19081493

**Published:** 2026-04-08

**Authors:** Fangyuan Gong, Yiming Yao, Hongkuan Li, Yuanping Xu

**Affiliations:** 1 School of Civil and Transportation Engineering, Hebei University of Technology, 5340 Xiping Road, Beichen District, Tianjin 300401, China; 202331605046@stu.hebut.edu.cn; 2 Huitong Construction Group Co., Ltd., No. 69, Shiji East Road, Baoding 074000, China; m18833244184@163.com (H.L.); 18630206864@163.com (Y.X.)

**Keywords:** steel fiber, I-optimal, mechanical properties, cement concrete

## Abstract

This study systematically investigates the influence of steel fibers on the mechanical properties of cement concrete. End-hook, shear, and milling type steel fibers were selected, with comparisons made to copper-plated and corroded steel fibers. The effects of fiber type, aspect ratio (40–60), and volume content (0.5–1.5%) on the compressive, flexural, and splitting tensile properties of concrete were analyzed. A multi-objective mechanical performance prediction model was established using a combined macro- and micro-scale testing approach, integrated with response surface methodology (RSM) and I-optimal design. The results indicate that steel fibers can significantly enhance the overall mechanical properties of concrete. Among the types tested, the end-hook fiber exhibited the best performance in compressive and splitting tensile strength, and the 28-day compressive strength increased by 41% compared with plain concrete, while the milling fiber showed the greatest improvement in flexural strength, and the value reached up to 72%. Furthermore, the failure mode observations indicated that steel fiber incorporation fundamentally altered the fracture behavior of concrete, transitioning it from brittle fracture to quasi-ductile behavior with post-crack load-carrying capacity, particularly for end-hook and milling fiber types. An optimal parameter window for the fiber reinforcement effect was identified, with the best comprehensive performance achieved at an aspect ratio of 50–60 and a fiber content of 0.5–1.0%. The enhancement effect of copper-plated and corroded steel fibers was limited due to reduced interfacial bonding performance. The developed model demonstrates high prediction accuracy, providing a theoretical and experimental basis for the engineering application of fiber-reinforced concrete.

## 1. Introduction

As the core material of modern infrastructure construction, cement concrete has been widely used in the world because of its excellent compressive performance, easy forming and economy [1,2,3]. As the world’s largest cement producer, China is more dependent on it in infrastructure construction. However, the inherent brittleness and low tensile strength of concrete materials make it easy for cracks to form during service. These cracks continue to expand under the combined action of load and environment, which not only significantly weakens the bearing capacity and durability of the structure, but also brings high maintenance costs and even causes catastrophic accidents in extreme cases, which seriously threatens public safety.

In order to effectively improve the crack resistance and toughening performance of concrete, fiber reinforcement technology has become one of the most promising modification methods in engineering applications [4]. Among available reinforcement strategies, fiber-reinforced concrete has emerged as the most extensively studied composite system, with comprehensive reviews documenting performance improvements across compressive, tensile, and flexural responses for both steel and synthetic fiber types [5]. By uniformly incorporating a large number of short and discrete fibers into the cement matrix, the technology uses the interfacial bonding force between the fiber and the matrix to form an effective ‘bridging’ stress at the tip of the micro-crack, thereby inhibiting the initiation and expansion of the crack, so that the concrete can still maintain a certain residual strength and significant deformability after cracking [6]. Among many fiber types, steel fiber can achieve efficient stress transfer and energy absorption due to its thermal expansion coefficient similar to that of concrete, its high elastic modulus, and its excellent tensile strength, thus occupying a dominant position in structural applications [7]. A large number of engineering practices [8,9] show that the incorporation of steel fiber can significantly improve the tensile, bending, impact and fatigue resistance of concrete. Critically, beyond these strength improvements, steel fibers are well known to substantially enhance the ductility and energy absorption capacity of concrete. Plain concrete fails in a sudden and brittle manner under tensile and flexural loading; fiber incorporation fundamentally changes this behavior by bridging cracks and sustaining residual load after first cracking, thereby enabling a quasi-ductile failure mode that is essential for structural safety.

The reinforcement effect of steel fiber on concrete is affected by its own geometric and physical parameters. The key parameters include the shape of the fiber (such as end-hook type, shear type, and milling type) [10], aspect ratio (ratio of length to diameter) [11], volume content [12] and surface state [13] (such as smooth, copper plating, or corrosion). These parameters directly determine the dispersion of the fiber in the mixture, the spatial distribution and orientation in the hardened body, and the interfacial bonding properties formed with the cement matrix. For example, the end-hook fiber enhances the mechanical bite force through the anchoring effect of the end [14]; single-fiber pull-out studies have further quantified the anchoring contribution of the hooked end, demonstrating that mechanical anchorage can account for more than 50% of total pull-out energy [15]; the aspect ratio affects the pull-out work and toughening efficiency of the fiber. In particular, the effects of aspect ratio on flexural and mechanical performance have been systematically evaluated across both recycled and conventional aggregate concrete [12], and the equivalency of corrugated and hooked fibers in bond strength has been established [16]. The volume content is directly related to the average spacing of the fibers and the number of bridging cracks [17,18,19,20,21]. These beneficial effects have been consistently demonstrated across a wide range of concrete strength grades, from normal- to ultra-high-strength matrices, with fiber dosage and length simultaneously exerting a synergistic influence on the tensile and flexural response [19]. In the domain of ultra-high-performance matrices, Yoo et al. [20] examined the interplay of fiber shape, aspect ratio, and volume fraction on flexural performance simultaneously, providing a multi-factor perspective; yet such systematic investigations remain scarce for normal-strength concrete, particularly when different fiber types are tested within a unified experimental framework.

The mechanical behavior of cracked concrete and the theoretical framework for describing it have been progressively established through decades of systematic research. Research on the fracture mechanics of concrete has undergone decades of development, accumulating extensive theoretical and experimental achievement [22]; the development of a series of theories and models (such as fiber spacing theory [23] and the equivalent crack model [24]) has gradually deepened the understanding of material fracture behavior. Systematic research of steel-fiber-reinforced concrete has also been carried out. From the early mechanism exploration to the later performance characterization and model establishment, rich results have been accumulated. Although Chinese scholars started relatively late in this field, they quickly kept up with the international pace through continuous experimental research [25], theoretical innovation and engineering practice, and formulated relevant design and construction procedures [26,27,28,29,30], which greatly promoted the standardized application of steel-fiber-reinforced concrete. In recent years, with the development of new cement-based materials such as ultra-high-performance concrete, steel fiber, as its key reinforcing component, has shown a broader application prospect [31]. Alongside these material-level advances, a parallel and increasingly prominent research trend has emerged: the integration of systematic experimental validation with data-driven predictive modeling. This combined paradigm has proven effective in capturing the complex, nonlinear relationships governing the mechanical behavior of fiber-reinforced cementitious systems. Shafaie et al. [32] demonstrated this approach by coupling push-out and slant shear tests with a fuzzy logic inference system to predict the interfacial bond strength of fiber-reinforced self-compacting concrete, achieving high predictive accuracy with a limited number of experimental runs. In the domain of steel-fiber-reinforced concrete (SFRC) property prediction, Kang et al. [33] integrated an extensive literature-based experimental database with machine learning algorithms to simultaneously predict compressive and flexural strengths, systematically quantifying the influence of fiber volume fraction and mix design parameters. Zheng et al. [34] further demonstrated that ensemble learning methods exhibit a distinct advantage in modeling the nonlinear flexural response of SFRC, with an optimal R^2^ of 0.96. Congro et al. [35] employed artificial neural networks to predict the residual flexural strength of fiber-reinforced concrete across multiple fiber and matrix parameters, with all regression coefficients exceeding 0.92. Beyond machine learning approaches, response surface methodology (RSM) has been widely validated as a statistical optimization tool for cementitious mix design, capable of modeling nonlinear multi-factor interactions with far fewer experimental runs than full factorial designs [36]. At the level of individual performance indices, RSM with D-optimal design has been applied directly to SFRC systems, simultaneously optimizing fiber volume fraction and aspect ratio to maximize splitting tensile strength and toughness under multi-objective constraints [37]. Despite these advances, existing modeling studies predominantly rely on aggregated published datasets and tend to focus on single mechanical performance indices, without systematically addressing the interaction effects among fiber shape, aspect ratio, and volume content within a unified experimental framework, nor targeting the simultaneous optimization of multiple mechanical properties.

Despite remarkable research outcomes, several important directions in current SFRC research require further exploration. First, as mentioned, the lack of studies on multi-parameter coupling effects makes it difficult to achieve the optimal balance between performance and cost in material design. Second, recent studies employing scanning electron microscopy (SEM) have introduced the concept of a Fiber Interfacial Transition Zone (FITZ)—a 30 μm wide strip around the fiber that is more susceptible to microcracking than the conventional aggregate–paste ITZ [38]—while nano-modification studies have shown that the pore structure and microhardness of the ITZ can be systematically improved [3]. The microstructure, composition of hydration products, chemical bonding state, and their evolution during stress in the ITZ are key to deeply understanding stress transfer, fiber pull-out, and damage mechanisms, yet related research is still insufficient. Additionally, comparative studies between different fiber materials are notably lacking. For example, the actual effectiveness of copper-plated steel fiber in improving corrosion resistance and the long-term effect of corroded steel fiber on interfacial properties are crucial for the scientific selection of fibers in engineering practice. Finally, existing mechanical performance prediction models often fail to fully consider key factors such as complex fiber shape, three-dimensional random distribution, and interface bond–slip constitutive relationships, resulting in limited model accuracy and generalizability.

To address the aforementioned challenges, this study aims to conduct systematic research. The core contents include: designing and implementing mechanical property tests for SFRC covering different fiber types (end hook, shear, and milling), different aspect ratios, and different volume contents, with comparisons to copper-plated and corroded steel fibers to comprehensively evaluate the characteristics and application potential of various fibers. In terms of theoretical modeling, a mechanical performance prediction model considering multiple factors (fiber type, aspect ratio, and content) will be constructed based on response surface methodology (RSM) and I-optimal experimental design. Multi-objective optimization of the mix proportion will subsequently be performed to identify the optimal combination of fiber parameters.

In summary, steel fiber reinforcement technology holds clear and significant value for improving the safety, durability, and sustainability of concrete structures. Through systematic experimentation and data modeling methods, this study aims to expand the existing research and provide solid theoretical support and feasible technical solutions for the efficient and precise application of steel-fiber-reinforced concrete in highway engineering and other infrastructure fields. This study focuses on quantifying the macroscopic effects of fiber parameters through experimental design and statistical modeling, and discusses the underlying mechanisms based on observed mechanical responses.

## 2. Raw Materials and Experimental Design

### 2.1. Raw Materials

#### 2.1.1. Aggregate

**Figure 1:**
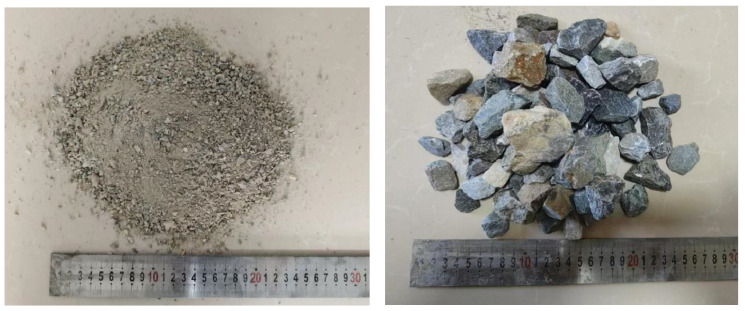
The surface state of coarse and fine aggregate.

The coarse aggregate used in this test is limestone, and the fine aggregate is river sand, which is provided by Huitong Road and Bridge Construction Group Highway Engineering Co., Ltd. (Gaobeidian City, China). The technical indicators of coarse and fine aggregates are shown in Table 1. The surface state of coarse and fine aggregate is shown in Figure 1.

#### 2.1.2. Fiber Materials

The fibers used in this experiment include end-hook steel fiber, shear steel fiber, milling steel fiber, and copper-plated end-hook steel fiber, which were provided by Hebei Baihang Engineering Co., Ltd. (Handan, China). In addition, self-made corroded end-hook steel fiber was also used in this experiment. The parameters of various fibers are shown in Table 2.

Through literature research and investigation, three types of steel fibers—end-hook type, shear type and milling type—were selected as the preparation materials, and three different aspect ratios were selected for each type. In terms of other fibers, corroded steel fibers and copper-plated fibers were selected as controls. The steel fiber density, chemical composition, tensile strength, bending performance and other indicators were optimized to meet the requirements of the specification. The technical indicators of various fibers are shown in Table 3, Table 4, Table 5 and Table 6.

**Figure 2:**
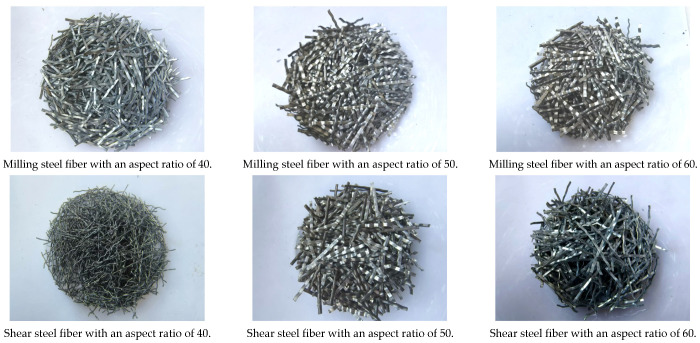

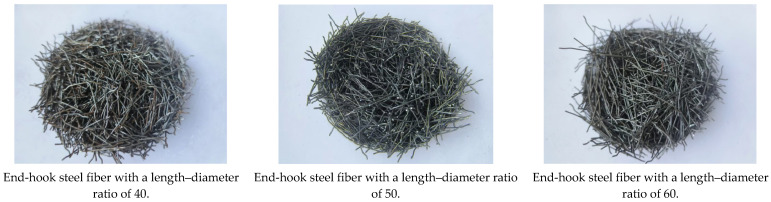
Different types of steel fibers with different aspect ratios.

Three types of steel fibers, namely end-hook type, shear type and milling type, were selected as the preparation materials, and three different aspect ratios of 40, 50 and 60 were selected for each type, as shown in Figure 2. In order to analyze the influence of fiber surface state on the crack resistance of fiber-reinforced concrete, the corroded steel fiber was prepared, and the best simple preparation conditions of corroded steel fiber (10% NaCl solution, soaking for 10 days) were optimized by setting the control group. A number of corroded steel fibers were prepared according to this condition, which were in contrast with copper-plated fibers and ordinary steel fibers.

**Figure 3:**
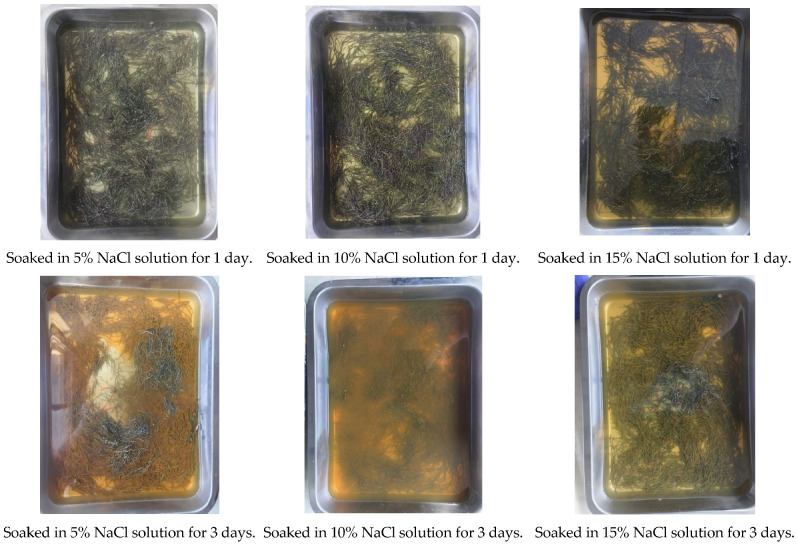

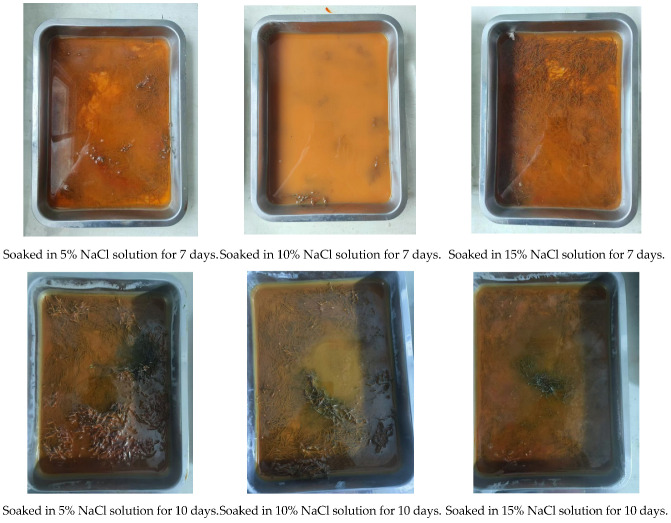
The corrosion process of steel fiber under different NaCl concentrations.

**Figure 4:**
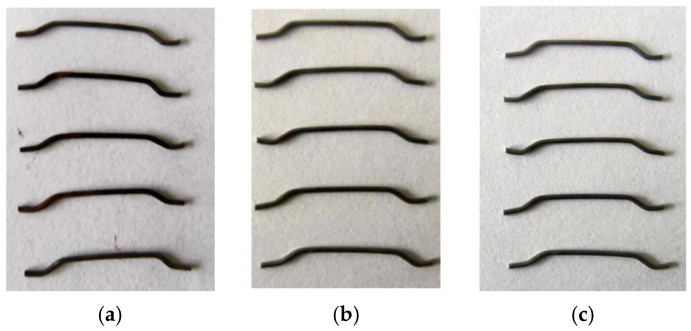
Corrosion effect of different concentrations of NaCl solution for 10 days: (**a**) 5%; (**b**) 10%; (**c**) 15%.

In order to optimize the best corrosion conditions and change the surface state of the fiber to form a contrast effect with the surface state of ordinary steel fiber and copper-plated fiber, ordinary steel fiber was soaked in different salt water concentrations of 5%, 10% and 15%, and compared with the evaporation group without steel fiber in the same natural environment, as shown in Figure 3. After 10 days’ treatment, the mass changes of end-hook steel fiber immersed in different salt water concentrations of 5%, 10% and 15% were 7.5 g, 11 g and 7 g, respectively, indicating that the corrosion effect of steel fiber is best under 10% salt water concentration, and the corrosion effect of NaCl solution with different concentrations for 10 days is shown in Figure 4; thus, this condition was selected to prepare corroded steel fiber.

**Figure 5:**
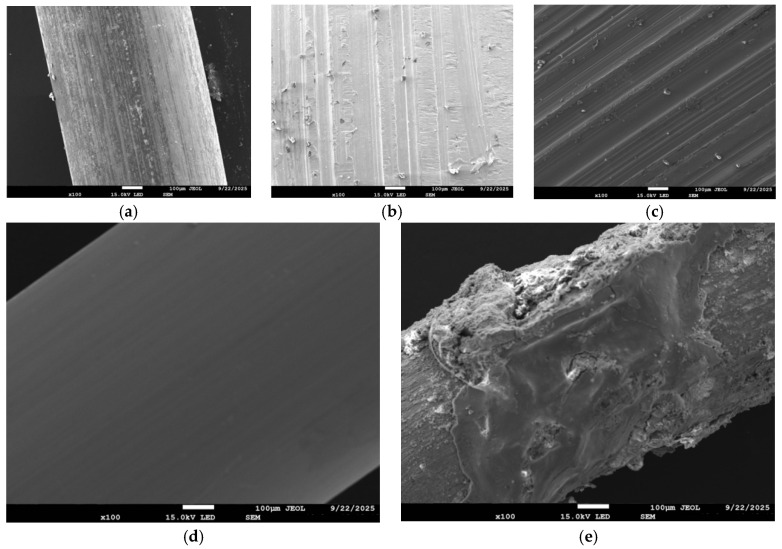
The micro-surface morphology of different types of steel fibers before incorporation: (**a**) end-hook steel fiber; (**b**) shear steel fiber; (**c**) milling steel fiber; (**d**) copper-plated steel fiber; (**e**) corroded steel fiber.

Figure 5 shows the micro-surface morphology of different types of steel fibers before incorporation. It can be clearly observed from the figure that there are significant differences in the initial surface state of different fibers. The surface of the hooked steel fiber is relatively clean, and the longitudinal lines formed by the cold drawing process are relatively dense. Milled steel fibers and shear steel fibers have highly rough and irregular textures, which are considered to provide stronger physical meshing and interfacial friction when combined with the cement matrix. In contrast, the surface of copper-plated steel fiber is very smooth and homogeneous. The surface of the corroded steel fiber is not a dense steel body but is covered by a layer of loose and porous corrosion products, which has poor structural integrity.

#### 2.1.3. Cementitious Materials

**Figure 6:**
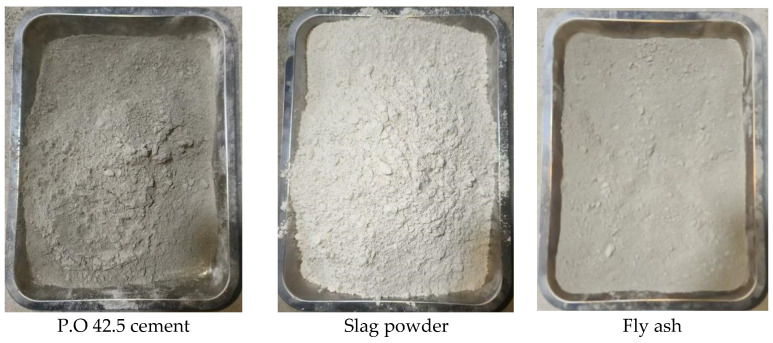
Morphology of cementitious materials.

Among the cementing materials used in the experiment, the cement is P.O 42.5 ordinary Portland cement produced by Jinyu Jidong Cement Group Co., Ltd. (Hengshui, Hebei, China), the fly ash is secondary fly ash, and the slag powder is S95 slag powder. The performance parameters are shown in Table 5, Table 6 and Table 7. The morphology of the cementitious material is shown in Figure 6.

### 2.2. Mix Proportion Design

Based on the discontinuous grading design (coarse aggregate: 10–20 mm single-grain limestone crushed stone) and the principle of slurry volume optimization, the water–binder ratio was strictly controlled at 0.36, which meets the requirements of low hydration heat and high compactness of high-strength concrete. The total content of fly ash and slag powder in the cementitious system is 36%, and the strength and impermeability in the later stage are improved by the pozzolanic effect and micro-aggregate filling. The sand ratio is 46.2%, which is significantly higher than Specification for Mix Proportion Design of Ordinary Concrete (JGJ 55-2011) [39] to compensate the gap defect of single-grain coarse aggregate grading. The benchmark matching was determined through system trial matching, as shown in Table 8.

### 2.3. Grading Design

The performance of cement stabilized macadam is significantly affected by gradation structure. The skeleton dense structure has both a coarse aggregate skeleton and a fine aggregate filling function, and has excellent stability and crack resistance, so this structure is adopted in the experiment. The gradation design of steel fiber cement concrete should consider the distribution and orientation of steel fiber and its bond with the matrix in order to exert the effect of strengthening and toughening. Therefore, the maximum particle size of coarse aggregate is limited to 10~20 mm in this study. This restriction is mainly based on two aspects: firstly, to prevent large aggregates from hindering the uniform dispersion and three-dimensional orientation of steel fibers, to prevent fibers from “bridging” or gathering, and to weaken the crack resistance; secondly, smaller aggregate helps to improve the workability of fiber-reinforced concrete and reduce segregation. At the same time, the maximum aggregate particle size is less than two-thirds the length of the steel fiber to ensure effective function.

On this basis, the discontinuous grading design is adopted, and the coarse aggregate is 10~20 mm single-particle limestone macadam. According to Pebble and Crushed Stone for Construction (GB/T 14685-2022 [40]), the sand ratio is increased to 45%, and coarse sand with fineness modulus of 2.9 is selected to compensate for the increase in porosity caused by discontinuous gradation. This design forms a stable skeleton with mortar as the continuous phase and coarse aggregate as the dispersed phase, which fully wraps the coarse aggregate and steel fiber, ensures the bonding strength of fiber–matrix interface, improves the cohesion of mixture and reduces the risk of fiber agglomeration. The grading design follows Standard for Design of Steel Fiber Reinforced Concrete Structures (JGJ/T 465-2019) [30], and the specific grading is shown in Table 9.

### 2.4. Specimen Preparation and Experimental Scheme

#### 2.4.1. Specimen Preparation

**Figure 7:**
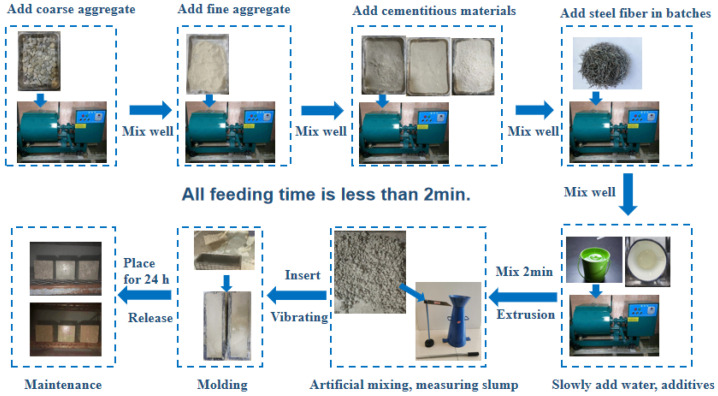
Flow chart of steel fiber cement concrete mixing.

Cube specimens (100 mm × 100 mm × 100 mm) were used for compressive and splitting tensile strength tests. Prism specimens (100 mm × 100 mm × 400 mm) were used for flexural strength and toughness tests. All specimens were prepared by vibration compaction in accordance with the Test Methods of Cement and Concrete for Highway Engineering (JTG 3420-2020) [41]. The mixing process for steel fiber concrete is illustrated in Figure 7.

According to the designed aggregate gradation and test scheme, a series of test steps, such as batching, mixing, oil brushing, charging and tamping, were carried out, respectively. Finally, the specimens were placed on a vibrating table together with the test formwork for compaction, and after a period of time, the formworks were removed and moved to the standard curing room for curing.

#### 2.4.2. Indoor Experimental Design

A three-factor, multi-level experimental design was employed to systematically study the effects of aspect ratio (Factor A), volume content (Factor B) and fiber type (Factor C) on the mechanical properties of concrete.

Factor A (aspect ratio): Three levels: small (S, 40), medium (M, 50), and large (L, 60). (Copper-plated and corroded fibers used a single aspect ratio of 60.)

Factor B (volume content): Three levels: 0.5%, 1.0%, and 1.5%.

Factor C (fiber type): Five types, including end-hook (DS), shear (SS), and milling (MS) steel fibers, as well as copper-plated (C) and corroded (X) steel fibers.

The experimental group should be composed of one reference group and 45 (5 × 3 × 3) fiber-reinforced concrete groups. However, considering that copper-plated steel fiber and corroded steel fiber are used as the research contrast of surface properties, the core research goal is to verify the independent influence of steel fiber surface state on interfacial bonding properties. In order to avoid the redundancy of variables, only one aspect ratio of these two fibers was selected as the experimental material. Finally, one group of plain concrete experiments and 33 groups of fiber concrete experiments were carried out, and each group underwent cube compressive, flexural and splitting tensile experiments, respectively. Among the fiber types, DS is end-hook steel fiber, SS is shear steel fiber, MS is milling steel fiber, C is copper-plated steel fiber and X is corroded steel fiber. In the aspect ratio of fiber, S is small aspect ratio, M is medium aspect ratio and L is large aspect ratio.

#### 2.4.3. Experimental Plan

Mechanical property tests were conducted on a total of 318 compressive specimens, 159 flexural specimens, and 318 splitting tensile specimens after standard curing to the specified ages (7 and 28 days). The compressive strength, splitting tensile strength and flexural tensile strength of the specimens were tested according to the Standard of Mechanical Properties Test Method of Ordinary Concrete (GB/T 50081-2019) [42].

**Figure 8:**
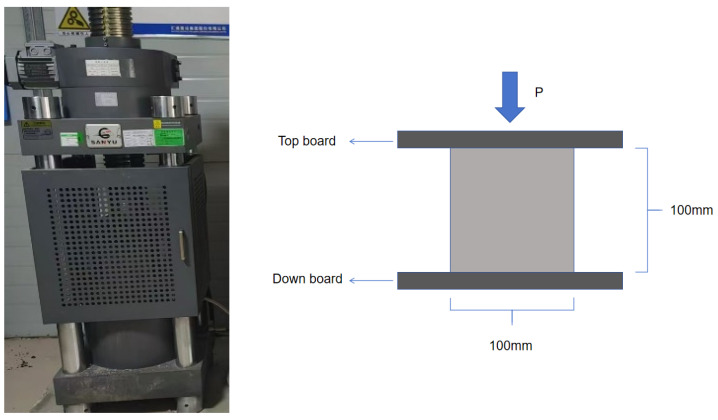
Cube compressive experimental instrument and schematic diagram.

**Figure 9:**
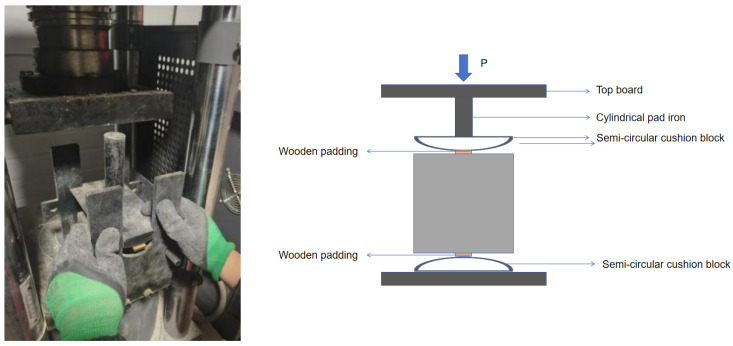
Cube splitting tensile test instrument and schematic diagram.

The cube compressive strength and splitting tensile strength tests were all completed by a micro-electro-hydraulic servo pressure testing machine (HYE-2000, Sanyu Machinery Equipment Co., Ltd. Yantai, China), and the experimental instruments are shown in Figure 8 and Figure 9 below.

The compressive strength test adopts a 100 mm × 100 mm × 100 mm cubic nonstandard specimen. The obtained data were multiplied by the size conversion coefficient of 0.9 according to the Standard of Test Method of Fiber Concrete (CECS 13-2009) [27] to convert it into the standard cubic compressive strength. The splitting tensile strength test also adopts a 100 mm cube specimen, and the test results are multiplied by the size conversion coefficient of 0.85 according to the Standard for Test Methods of Fiber Concrete (CECS 13-2009) [27].

In the experiment, a central beam specimen with the size of 100 mm × 100 mm × 400 mm was used. In order to ensure the accuracy of the test data, a press with a small range was used for the bending and tensile strength test. The curing age was 7d and 28d, and the specimen was taken out of the room after the curing period had ended. Prior to testing, the surface moisture of each prism specimen was removed using a damp cloth. The specimen was then positioned on the support frame with an overhang of 50 mm at each end, yielding an effective span of 300 mm between the two supports. This 300 mm span was subsequently divided into three equal segments of 100 mm each by marking two lines on the specimen surface using colored chalk. The two loading points of the indenter were aligned with these two marked lines, consistent with the four-point bending configuration specified in CECS 13-2009 [27].

(1) $$f_{f}=\frac{Fl}{bh^{2}}$$

where *f*_f_ is the flexural strength of concrete, in MPa; *F* is the load when the specimen is damaged, in n; *l* is the span between supports, in mm; *b* is the section width of the specimen, in mm; and *h* is the section length of the specimen, in mm.

According to the standard [27], the final flexural strength should be obtained by multiplying the data by the size conversion coefficient of 0.8.

## 3. Test Results and Analysis of Mechanical Properties

In this study, a total of 34 distinct fiber-reinforced concrete mixtures, including one plain concrete reference group, were designed and tested. For each mechanical property—cube compressive strength, flexural strength, and splitting tensile strength—three replication specimens were made and tested for each mixture. The strength value for each mixture was calculated in accordance with GB/T 50081–2019 [42]: the arithmetic mean of the three measurements was reported as the representative strength if the difference between any individual test result and the median of the three replicates did not exceed 15% of the median; if not, the median value was adopted. All results are expressed as measured representative intensity values, with the standard deviation of each dataset shown as error bars in the figures.

### 3.1. Cube Compressive Strength

In this study, the effects of fiber type, aspect ratio, and volume content on the cube compressive strength of cement concrete were systematically investigated. The results indicate that fiber incorporation significantly influences compressive strength, with the degree of enhancement or reduction depending on the synergistic interaction between fiber material, geometry, and content. Ordinary steel fibers generally exhibited a good reinforcing effect. In contrast, copper-plated and corroded steel fibers performed poorly within the tested dosage range, even leading to a deterioration in the matrix strength.

**Figure 10:**
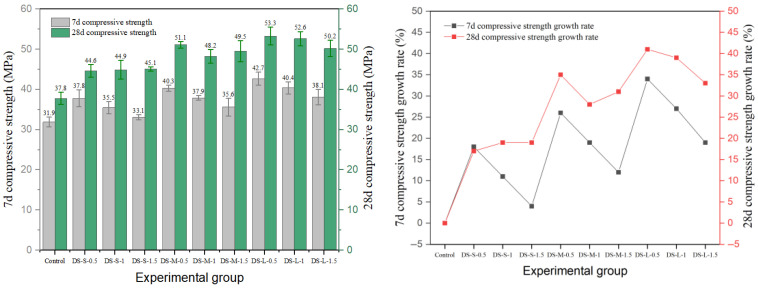
Compressive strength and strength growth rate of hooked-steel-fiber-reinforced cement concrete.

Among the three kinds of steel fibers, the reinforced effect of end-hook steel fiber on cement concrete is more prominent, as shown in Figure 10, and its 28-day compressive strength is the highest among all fiber groups. The 28-day strength of the DS-L-0.5 group reached 53.3 MPa, which was 41% higher than that of the reference group (37.8 MPa). This level of enhancement is consistent with the range reported in the literature: Zhang et al. [1] found that hooked-end fibers with 1.0–1.5% content increased compressive strength by 4–24% in normal-strength concrete, while the effect is amplified in higher-strength matrices due to improved fiber–matrix compatibility. The reinforcement effect of end-hook fiber is highly sensitive to the dosage. Under each aspect ratio, 0.5% dosage usually corresponds to the highest 7-day strength. When the content increased to 1.0%, the 28-day strength decreased slightly; although the strength increased to 1.5%, it did not exceed the peak value of the 0.5% dosage group. This shows that excessive fibers may introduce local defects and weaken the compactness of the matrix, thus offsetting the reinforcement effect. This inversely correlated trend at higher dosages has been corroborated by multiple prior studies, wherein compressive strength reached a plateau or even decreased beyond a 1.0–1.5% fiber volume fraction due to fiber agglomeration and matrix discontinuity [1,9]. From the long-term performance, the end-hook fiber significantly promoted later strength growth: the strength growth rate of the control group was 18% from 7 days to 28 days, while that of the end-hook fiber group was generally between 25% and 39%, which reflected its positive role in bridging micro-cracks and delaying damage accumulation.

**Figure 11:**
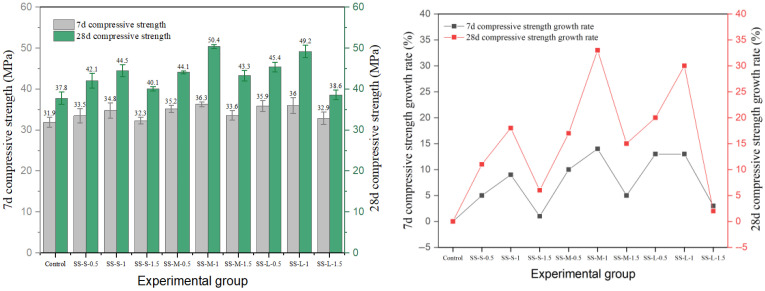
Compressive strength and strength growth rate of shear-steel-fiber-reinforced concrete.

**Figure 12:**
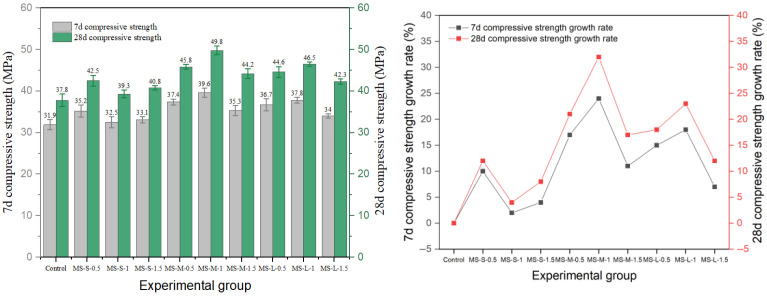
Compressive strength and strength growth rate of milled-steel-fiber-reinforced cement concrete.

Shear type and milling steel fiber also have an obvious reinforcement effect, and the experimental results are shown in Figure 11 and Figure 12. The results show that the optimal parameter combination of shear-steel-fiber-reinforced concrete is different from that of milling-steel-fiber-reinforced concrete, and the shear steel fiber reaches the peak strength of 50.4 MPa at the medium length–diameter ratio and 1.0% content (SS-M-1). The strength of milled steel fiber changes more smoothly, and the peak strength of the medium length–diameter ratio group (MS-M-1) is 49.8 MPa, and the strength decreases by 11.2% after exceeding the optimal dosage, which is smaller than that of end-hook fiber and shear fiber, indicating that its surface texture enhances the mechanical bite force and is evenly distributed, with little damage to the continuity of the matrix.

**Figure 13:**
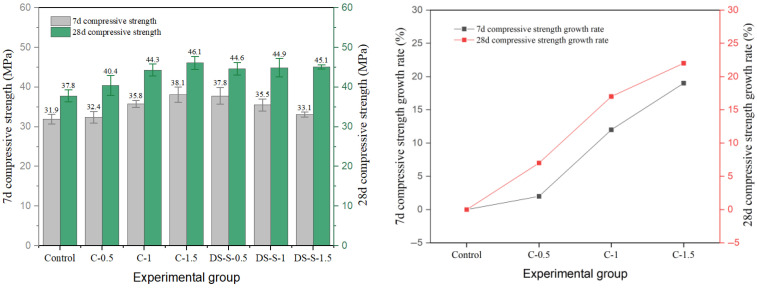
Compressive strength and strength growth rate of copper-plated steel-fiber-reinforced cement concrete.

**Figure 14:**
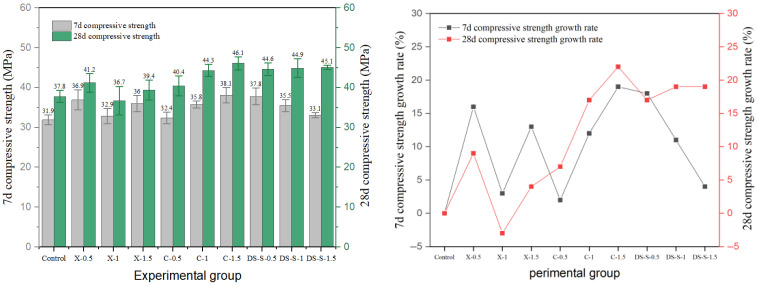
Compressive strength and strength growth rate of corroded-steel-fiber-reinforced cement concrete.

The experimental results of copper-plated steel-fiber-reinforced and corroded steel-fiber-reinforced are shown in Figure 13 and Figure 14. The performance of corroded steel fibers was even poorer, showing only a slight 9% increase at the 0.5% dosage (X-0.5). At higher dosages, the strength fell below that of the reference group. Furthermore, the later-age strength gain rate for all corroded fiber groups was significantly lower than that of the control group. Corrosion products disrupted the integrity of the fiber–matrix interface, hindered effective bonding, and may have inhibited cement hydration.

In summary, steel fibers can significantly improve the compressive strength of concrete, but their reinforcing effect is confined to an optimal window of content and aspect ratio. The enhancement effect of copper-plated and corroded steel fibers was limited due to interfacial issues. Fiber incorporation generally promoted later-age strength gain, demonstrating its positive role in bridging micro-cracks and enhancing long-term performance.

### 3.2. Flexural Strength

**Figure 15:**
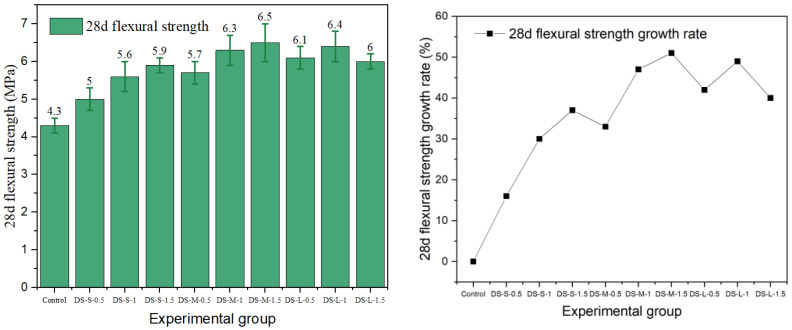
Flexural strength and strength growth rate of hooked-steel-fiber-reinforced cement concrete.

**Figure 16:**
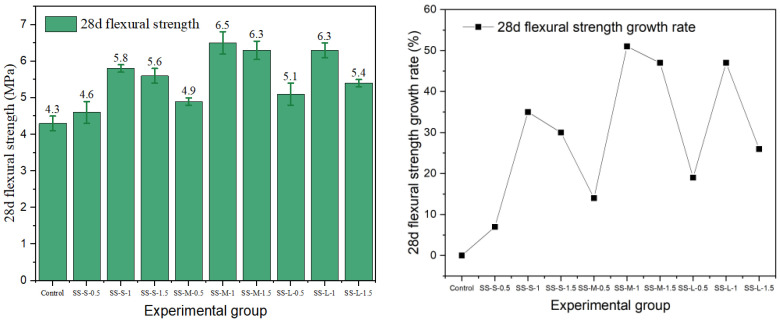
Flexural strength and strength growth rate of shear-steel-fiber-reinforced cement concrete.

**Figure 17:**
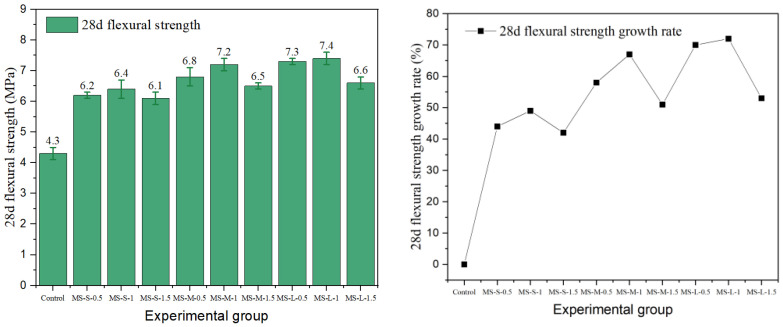
Milling-steel-fiber-reinforced cement concrete flexural strength and strength growth rate.

**Figure 18:**
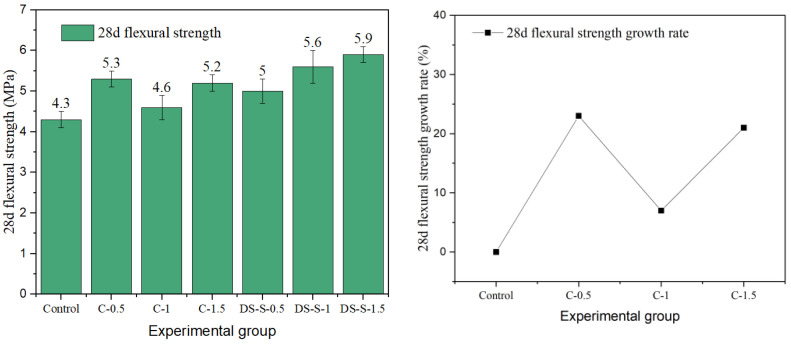
Flexural strength and strength growth rate of copper-plated steel-fiber-reinforced cement concrete.

**Figure 19:**
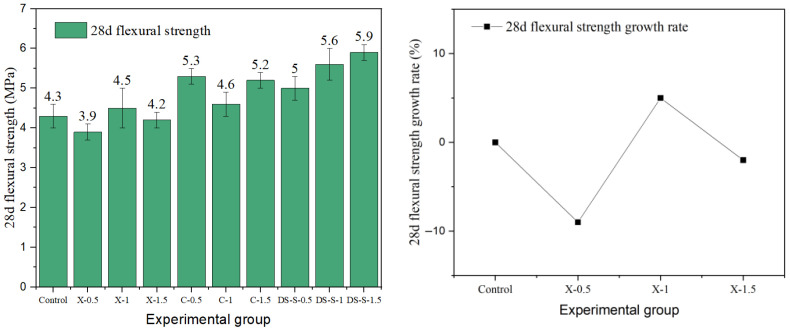
Flexural strength and strength growth rate of corroded-steel-fiber-reinforced cement concrete.

Flexural strength is a key index for evaluating the crack resistance and toughness of fiber-reinforced concrete. In this study, the effects of different fiber types and the parameters on the flexural properties of cement concrete were systematically investigated. The results are presented in Figure 15, Figure 16, Figure 17, Figure 18 and Figure 19.

The data show that the addition of fibers can significantly alter the flexural properties of concrete, but the toughening effect is highly dependent on fiber geometry, surface characteristics, aspect ratio, and volume content. Generally, milling and end-hook type steel fibers were most effective in improving flexural strength, while other fibers exhibited limited or even negative reinforcing effects due to the inherent limitations.

The flexural performance of end-hook steel fiber relied primarily on its end-anchorage mechanism. Its optimum flexural strength was achieved in the medium aspect ratio group with 1.5% content (DS-M-1.5), reaching 6.5 MPa with a 51% increase. The anchoring mechanism of the hooked end has been independently quantified by pull-out experiments showing that the hook contributes 50–57% of total pull-out energy [15], which directly underpins the flexural bridging capacity observed in this study. The end hook provides a mechanical interlock during fiber pull-out, effectively delaying crack propagation. Unlike the compressive behavior, the flexural strength of the composite increased continuously with fiber content within the tested range. This indicates that the anchoring effect can be fully utilized at higher contents. However, in the large-aspect-ratio group, strength decreased at the 1.5% dosage, suggesting that the combination of excessive fiber length and high content may impair dispersion uniformity.

Milling steel fiber exhibited the best flexural performance overall. Its 28-day flexural strength peaked in the MS-L-1 group at 7.4 MPa, which was 72% higher than the control group (4.3 MPa). This magnitude of improvement is supported by recent comparative studies: Zhao et al. [43] demonstrated that milled fibers consistently outperformed hooked fibers in flexural performance across varying aspect ratios, attributing this advantage to the stronger mechanical interlock generated by their rough surface texture. This is attributed to its rough surface morphology, which enhances mechanical interlock and friction at the fiber–matrix interface, thereby providing higher bridging stress during crack propagation. The flexural strength increased with aspect ratio, with the large-aspect-ratio groups showing the best performance across all dosage levels. Its strength varied moderately with content, indicating that milling fiber can effectively realize its toughening potential even at lower contents.

The flexural performance of shear steel fiber was particularly sensitive to content. The strength of the best-performing group (SS-M-1) was 6.5 MPa, but strength decreased in all groups when the dosage increased to 1.5%. Due to the lack of end anchorage or distinct surface texture, it relies primarily on chemical bonding with the cement paste, resulting in a relatively weaker interface. At high contents, the straight fibers are prone to agglomeration, forming defects that disrupt stress transfer and lead to a decline in the reinforcing effect.

The flexural properties of copper-plated and corroded steel fibers were severely limited by interfacial issues. The copper-plated fiber achieved a strength of 5.3 MPa at 0.5% content. However, as the content increased, agglomeration induced by the smooth surface caused strength gain to stagnate, with a maximum increase of only 23%. The performance of corroded fiber was even poorer. The strength of the best group (X-1) was only 4.5 MPa, a mere 5% increase, while the strength of the other groups was even lower than that of the reference concrete. The loose oxide layer formed by corrosion products creates a weak transition zone at the interface, significantly weakening the fiber bridging effect.

Beyond the quantitative strength data reported above, the failure modes observed during the four-point bending tests provided important qualitative evidence of the ductility improvement imparted by steel fiber incorporation. Plain concrete specimens failed in a sudden and brittle manner: upon reaching peak load, a single dominant crack propagated rapidly through the cross-section, resulting in immediate and complete separation of the specimen with no residual load-carrying capacity. In contrast, all fiber-reinforced specimens exhibited a markedly different fracture behavior. After first cracking, the bridging fibers continued to transfer stress across the crack faces, preventing catastrophic separation and allowing the specimens to sustain a measurable residual load over considerably larger mid-span deflections before eventual failure by fiber pull-out or rupture. This transition from brittle fracture to quasi-ductile behavior was most evident in the milling and end-hook fiber groups, consistent with their superior interfacial bonding mechanisms; the rough surface texture of milling fibers and the mechanical anchorage of end-hook fibers both enhance the fiber–matrix interaction during crack opening, thereby prolonging energy dissipation. Shear steel fiber groups also demonstrated improved post-crack integrity compared to plain concrete, though the absence of mechanical anchorage made the fibers more susceptible to pull-out at higher deflections. The copper-plated and corroded fiber specimens showed the least improvement in post-crack behavior, with specimens exhibiting relatively abrupt load drops after peak, consistent with the weak interfacial bonding that also limited their strength performance. These observations confirm that the incorporation of steel fibers represents not merely a strength enhancement strategy but a fundamental improvement in the fracture ductility of concrete, a characteristic of critical importance for structural applications subjected to impact, seismic, or fatigue loading.

In summary, steel fibers can significantly improve the flexural strength and toughness of concrete through mechanisms such as mechanical interlock and end anchorage, with the milling type exhibiting the best overall performance. The enhancement provided by copper-plated and corroded fibers was limited by interfacial bonding. Unlike the trend for compressive strength, flexural strength generally increased with fiber content. This indicates that a higher fiber proportion can more effectively bridge macro-cracks and fully utilize the crack-resistance potential under flexural and tensile stresses.

### 3.3. Splitting Tensile Strength

**Figure 20:**
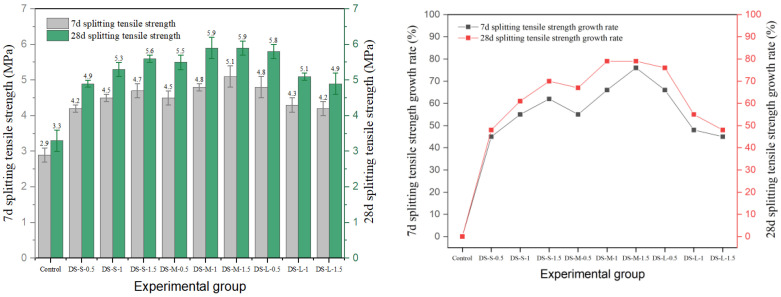
Splitting tensile strength and strength growth rate of hooked-steel-fiber-reinforced cement concrete.

**Figure 21:**
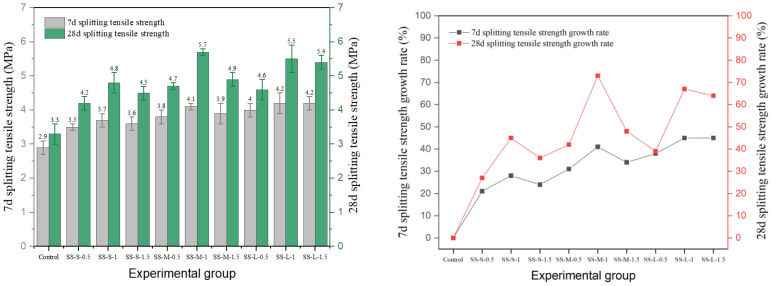
Splitting tensile strength and strength growth rate of shear-steel-fiber-reinforced cement concrete.

**Figure 22:**
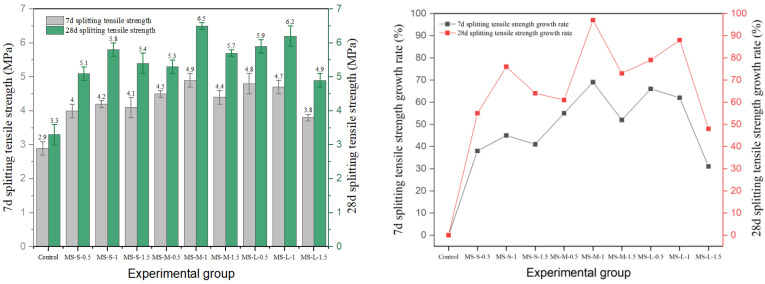
Splitting tensile strength and strength growth rate of milling-steel-fiber-reinforced cement concrete.

**Figure 23:**
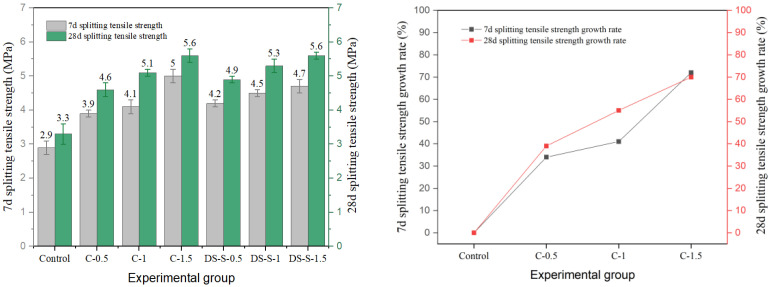
Splitting tensile strength and strength growth rate of copper-plated steel-fiber-reinforced cement concrete.

**Figure 24:**
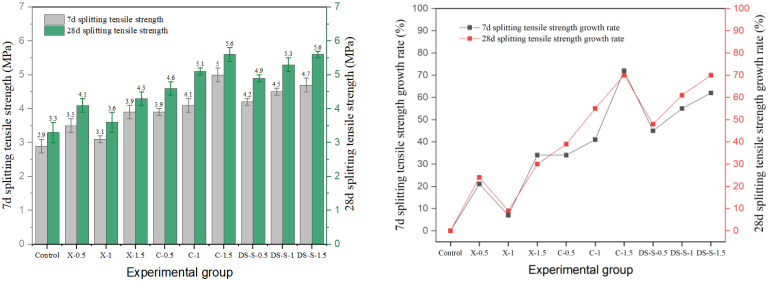
Splitting tensile strength and strength growth rate of corroded-steel-fiber-reinforced cement concrete.

In this study, the effects of different types of fibers on the splitting tensile strength of cement concrete are systematically investigated. The results are shown in Figure 20, Figure 21, Figure 22, Figure 23 and Figure 24, and the data show that there are significant differences in fiber reinforcement effects, which mainly depend on the mechanical properties, geometric characteristics of fibers and their interfacial bonding properties with the cement matrix.

Regarding splitting tensile strength, steel fibers exhibited an excellent reinforcing effect. The 28-day splitting tensile strength of end-hook steel fiber increased regularly with both content and aspect ratio. The strength of the medium-aspect-ratio group (DS-M-1.5) reached 5.9 MPa, representing a 79% increase over the reference group’s strength of 3.3 MPa. It is noteworthy that the splitting tensile strength continued to increase with fiber content, contrasting with the “optimal content” phenomenon observed in the compressive tests. This indicates that the end-anchorage effect is more fully mobilized under tensile stress.

Milling steel fiber performed best. The 28-day strength of the medium-aspect-ratio group (MS-M-1) reached 6.5 MPa, the highest value among all experimental groups and a 97% increase compared to the reference group. This is attributed to the strong mechanical interlock provided by the milled surface texture, which ensures effective stress transfer. However, when the content exceeded 1.0%, strength generally declined, indicating that the enhancement effect still exhibits an optimal threshold in terms of content.

The performance of shear steel fiber showed obvious sensitivity to content. In the medium-aspect-ratio group, peak strength (5.7 MPa) was achieved at 1.0% content (SS-M-1) and decreased to 4.9 MPa when the content increased to 1.5%. This inverted U-shaped trend stems from the straight fiber morphology, which is prone to agglomeration at high contents, leading to decreased stress transfer efficiency. In terms of strength development, shear steel fiber significantly promoted later-age strength gain. The strength gain rate from 7 to 28 days for the SS-M-1 group reached 39%, far exceeding the 14% of the reference group.

The splitting tensile strength of copper-plated steel fiber exhibited an initial increase followed by a decrease. At 1.5% content, the highest strength reached 5.6 MPa; however, the improvement was significantly lower than that achieved with ordinary steel fibers. The smooth copper-plated surface reduces the inter-fiber friction coefficient, leading to severe agglomeration when the content exceeds a critical value. The later-age strength gain rate of the C-1.5 group was only 12%, substantially lower than that of other dosage groups.

The behavior of corroded steel fiber was more complex, with strength showing non-monotonic changes. The strength was 4.1 MPa for the X-0.5 group, dropped to 3.6 MPa for X-1, and then rose to 4.3 MPa for X-1.5. This fluctuation stems from the dual effect of corrosion on the interface: moderate corrosion increases surface roughness and enhances mechanical interlock, whereas excessive corrosion causes the oxide layer to spall, creating a weak zone. The degradation of the fiber–matrix bond by corrosion products is consistent with findings from UHPC studies, where pre-corroded steel fibers caused a reduction in interfacial bond strength due to the formation of a loose, porous oxide layer at the fiber surface [2]. The dual effect of moderate corrosion increasing surface roughness while excessive corrosion forming a weak zone, observed in this study, was also noted in single-fiber pull-out investigations [18]. The later-age strength gain rate of all corroded fiber groups was lower than that of the reference group, indicating that interfacial damage continued to develop under long-term loading.

In summary, steel fibers, especially the end-hook and milling types, can significantly improve the splitting tensile strength of concrete. The enhancement is primarily achieved through end anchorage and the mechanical interlock provided by surface texture. The enhancement effect of copper-plated and corroded steel fibers was limited due to interfacial problems. The results of this study provide an important basis for the material design and engineering application of fiber-reinforced concrete in tensile-stress-dominated environments.

## 4. Mix Proportion Optimization Design and Model Establishment

### 4.1. Response Surface Methodology (RSM) and CCD Experimental Design

#### 4.1.1. Experimental Design

In this study, a three-factor, three-level central composite design (CCD) within the response surface methodology (RSM) framework was implemented using Design-Expert 13 software. The model responses were the cube compressive strength, flexural strength, and cube splitting tensile strength. The experimental design matrix and results are presented in Table 10, where Factor A represents the steel fiber aspect ratio, Factor B the volume content, and Factor C the fiber type.

Based on the data in Table 10, quadratic polynomial regression models for the various strength responses were developed using Design-Expert 13 software, as shown in Equations (2)–(6).

(2) $$\begin{array}{l} Y_{1}=38.90+1.42A-1.54B+1.65C_{1}-1.39C_{2}-0.2833AB+1.04AC_{1}-0.9056AC_{2}\\ -0.7889BC_{1}+0.3944BC_{2}-1.99A^{2}-1.92B^{2} \end{array}$$

(3) $$\begin{array}{l} Y_{2}=50.22+2.47A-0.7B+2.86C_{1}-1.22C_{2}-0.3583AB+1.89AC_{1}-1.22AC_{2}\\ +0.9667BC_{1}-0.7333BC_{2}-2.91A^{2}-3.26B^{2} \end{array}$$

(4) $$\begin{array}{l} Y_{3}=6.81+0.3A+1.778B-1.97C_{1}-0.4606C_{2}-0.1917AB+0.0333AC_{1}\\ -0.1667AC_{2}+0.0889BC_{1}+0.2722BC_{2}-0.414A^{2}-0.614B^{2} \end{array}$$

(5) $$\begin{array}{l} Y_{4}=4.66+0.1389A-0.0056B+0.2879C_{1}-0.3939C_{2}-0.175AB-0.1556AC_{1}\\ +0.1278AC_{2}+0.0889BC_{1}+0.0722BC_{2}-0.3202A^{2}-0.2202B^{2} \end{array}$$

(6) $$\begin{array}{l} Y_{5}=6.05+0.1556A+0.0722B+0.0879C_{1}-0.3758C_{2}-0.1917AB-0.1556AC_{1}\\ +0.1778AC_{2}-0.0389BC_{1}+0.1444BC_{2}-0.4474A^{2}-0.564B^{2} \end{array}$$

where *Y*_1_ is 7d compressive strength, *Y*_2_ is 28d compressive strength, *Y*_3_ is 28d flexural strength, *Y*_4_ is 7d splitting tensile strength, *Y*_5_ is 28d splitting tensile strength; A is the fiber aspect ratio, B is the fiber volume content (%), and *C*_1_ and *C*_2_ are dummy variables encoding the categorical factor of fiber type. When the fiber is hooked at the end, *C*_1_ = 0 and *C*_2_ = 0; when the fiber is shear type, *C*_1_ = 1 and *C*_2_ = 0; when the fiber is milling type, C_1_ = 0 and *C*_2_ = 1.

#### 4.1.2. Analysis of Variance

The analysis of variance (ANOVA) results for the CCD experiments is summarized in Table 11. According to the ANOVA results, the quadratic polynomial regression models for all five mechanical property indices were highly significant (*p*-value < 0.0001). Furthermore, the lack-of-fit *p*-value for each model was greater than 0.05, indicating that the models were well fitted, with no significant unexplained systematic variation, and that errors were primarily due to random factors.

Regarding the influence of individual factors, the sensitivity of each strength index varied. For the 7-day compressive strength, the steel fiber aspect ratio (A), volume content (B), and type (C) all had extremely significant effects (*p* < 0.01), with the order of influence (based on effect estimates) being content (B) > type (C) > aspect ratio (A). For the 28-day compressive strength, fiber type (C) had an extremely significant effect, and aspect ratio (A) a significant effect, while the effect of content (B) was not significant. For flexural strength, aspect ratio (A), content (B), and type (C) all exhibited highly significant influences, with the order of importance being A > C > B. For splitting tensile strength, fiber type (C) was a highly significant factor at both 7 and 28 days. The 7-day strength was also significantly affected by aspect ratio (A), whereas content (B) did not show a significant influence at either age.

Regarding model goodness-of-fit, the difference between the coefficient of determination (R^2^) and the adjusted R^2^ was less than 0.2 for all models, indicating good model reliability. Additionally, the adequate precision values for all models were substantially greater than 4, further confirming that the models had a good signal-to-noise ratio and predictive capability. This allows for effective navigation and optimization within the given factor space.

The study uses predicted R^2^ to evaluate the predictive ability of the model. Predicted R^2^ reflects the model’s ability to interpret new data. It can be seen from Table 11 that the difference between predicted R^2^ and adjusted R^2^ of all response variables is less than 0.2, which preliminarily indicates that the established model not only fits the existing data well but also has certain generalization prediction ability.

In summary, the established RSM models can reliably characterize the influence of steel fiber parameters (type, aspect ratio, and content) on the various mechanical properties of concrete. Based on these models, the optimal factor combinations for each individual response are presented in Table 12.

Table 12 lists the specific fiber parameter combinations and the corresponding predicted strength values required to maximize each mechanical property index individually. The results show that the optimal aspect ratio, volume content, and fiber type differ significantly depending on the specific strength property. Consequently, it is difficult for a single fiber parameter combination to simultaneously maximize all mechanical properties. Furthermore, the sensitivity of the different strength indices to the fiber geometric characteristics (aspect ratio and type) and content varies considerably.

Table 13 further gives the global optimal fiber parameter combination determined after comprehensively considering all five strength indices and its corresponding predicted strength values. Compared with the peak values of the individual strengths in Table 12, the predicted strength values under the comprehensive optimal conditions are all lower than the corresponding single optimal values, but the other mechanical indices are close except for the 28d flexural strength, which is greatly reduced due to the use of hook-end fibers. The comprehensive scheme provides a practical fiber reinforcement ratio for engineering application with multi-performance objectives in mind.

### 4.2. I-Optimal Optimization Method

Following the design and analysis of the CCD experiments using RSM, the statistical relationships between the factors and response values have been established in this study. It provides a foundational model for subsequent process optimization. The CCD incorporates a D-optimality criterion, which minimizes the generalized variance of the model parameter estimates by maximizing the determinant of the information matrix. However, high accuracy in parameter estimation cannot guarantee stability in prediction. Prediction stability is also a critical objective when developing a predictive model. Therefore, an I-optimal design within the RSM framework using Design-Expert 13 software was employed to achieve better model prediction performance in this study.

#### 4.2.1. Principle of the I-Optimal Method

Among various optimal design criteria, I-optimality (Integrated Variance Optimality) has garnered significant attention due to its advantage in minimizing prediction variance, particularly in scenarios where model prediction accuracy is the primary concern. The I-optimal design seeks to optimize the experimental design by minimizing the average prediction variance (APV) over a specified experimental region or region of interest (ROI). Mathematically, this is equivalent to minimizing the integrated variance of the predicted response across the region. Specifically, for a linear or polynomial regression model with parameter vector β, the variance of the predicted response at any point *x* is given by:

(7) $$Var(\hat{y}(x))=\sigma^{2}f^{T}(x)M^{-1}(\xi )f(x)$$

where *f*(*x*) is the regression vector at point *X*, and *M* − 1(*ξ*) is the information matrix derived from design *ξ*, *σ*^2^.

The I-optimal criterion seeks to minimize the integral of the prediction variance on the domain *χ*, which is expressed mathematically as:

(8) $$\min_{\xi }\int_{\chi }Var(\hat{y}(x))dx=\sigma^{2}\min_{\xi }\int_{\chi }f^{T}(x)M^{-1}(\xi )f(x)dx$$

This integral is equivalent to the weighting of the trace of the inverse of the information matrix on the integral of the outer product of the regression vector, that is:

(9) $$\min_{\xi }\mathrm{tr}(M^{-1}(\xi )B)$$

where the elements of matrix *B* are:

(10) $$\int_{\chi }f_{i}(x)f_{j}(x)dx$$

The core application of I-optimal design is in complex system optimization problems where accurate prediction over a region is more critical than precise parameter estimation. In this study on the mechanical properties of steel-fiber-reinforced concrete, the value of this method is particularly evident. When the primary goal is to accurately identify the optimal mix proportion (to maximize mechanical properties) within a multi-dimensional parameter space, it is essential to account for the interaction effects among key factors such as steel fiber type (end-hook, shear, and milling), aspect ratio (40–60), and volume content (0.5–1.5%).

For example, the experimental data of this study show that the peak flexural strength of end-hook steel fiber can reach 6.5 MPa under the medium length–diameter ratio and 1.5% dosage (DS-M-1.5), which is 51% higher than that of the control group, but its optimal compressive strength group (DS-L-1) is in a completely different parameter range. However, due to the surface texture characteristics, milled steel fiber shows a unique feature of slow decrease in splitting tensile strength optimization, and the strength only decreases by 12.3% when the content is 1.5%. This multi-objective, nonlinear response characteristic is suitable for building a high-precision prediction model through I-optimal design, and the global optimal solution can be achieved within the preset material performance target domain.

For the high cost and lengthy duration of fiber-reinforced concrete experimentation, I-optimal design enhances prediction reliability at critical performance thresholds by optimally allocating limited experimental resources across the design space. Compared to a CCD, which may require testing at least 27 parameter combinations, an I-optimal design can concentrate experimental efforts on sensitive regions and more accurately predict performance inflection points with a reduced number of trials. This approach can significantly reduce experimental cost and time while maintaining, or even improving, the overall prediction accuracy of the model.

Compared to the traditional D-optimality criterion, which focuses on precise parameter estimation by maximizing the information matrix determinant, the I-optimality criterion focuses on minimizing the average prediction variance across the region of interest. This makes it particularly suitable for fitting complex, high-dimensional models and provides more accurate predictions within the specified experimental domain, which is crucial for mixture design problems. The key differences between these two optimization criteria are summarized in Table 14.

#### 4.2.2. Experimental Design Results of the I-Optimal Method

The optimal design of response surface based on the I-optimal criterion is shown in Table 15. It shows that the I-optimal design generated by Design Expert 13 software in this study only needs 22 groups of experiments, which significantly reduces the demand for experimental resources and is a reduction from the 33 groups of experiments required by the CCD design. This simplification of the experimental amount stems from the optimization of the prediction variance of the design space by the I-optimal algorithm.

The statistical test results of the model are shown in Table 16, and the data confirm the effectiveness of the I-optimal design. The quadratic model of each response variable has passed significance verification (*p*-values of the model are all <0.05): 7d compressive strength (*p*_1_ = 0.0025), 28d compressive strength (*p*_1_ = 0.0037), 28d flexural strength (*p*_1_ = 0.0001), 7d splitting tensile strength (*p*_1_ = 0.0105) and 28d splitting tensile strength (*p* = 0.0156). At the same time, the *p*-value of the missing item for all models is >0.05, which indicates that the model error only comes from random variation, there is no systematic deviation, and the regression model has a high degree of fitting. Under the I-optimal optimization design, the predicted R^2^ of all models is positive, and the difference between the predicted R^2^ and the adjusted R^2^ is less than 0.2, which indicates that the model has not been over-fitted. It is worth noting that the I-optimal prediction R^2^ is basically the same as the CCD model, and some indicators are even better. This fully proves that the I-optimal design can ensure the prediction accuracy of the model.

Based on the above analysis, the mechanical properties prediction model of steel-fiber-reinforced concrete is finally established as follows (11)–(15):

(11) $$\begin{array}{l} Y_{1}=38.56+1.24A-1.43B+1.56C_{1}-1.50C_{2}-0.4756AB+1.40AC_{1}-0.7644AC_{2}\\ -0.5211BC_{1}+0.2935BC_{2}-1.23A^{2}-2.23B^{2} \end{array}$$

(12) $$\begin{array}{l} Y_{2}=50.23+2.24A-0.4336B+2.69C_{1}-1.45C_{2}-0.7685AB+2.87AC_{1}\\ -1.54AC_{2}+1.52BC_{1}-0.9541BC_{2}-2.46A^{2}-3.81B^{2} \end{array}$$

(13) $$\begin{array}{l} Y_{3}=6.88+0.2909A+0.1880B-0.2379C_{1}-0.5393C_{2}-0.2279AB+0.1068AC_{1}\\ -0.2159AC_{2}+0.1511BC_{1}+0.2368BC_{2}-0.4953A^{2}-0.6192B^{2} \end{array}$$

(14) $$\begin{array}{l} Y_{4}=4.67+0.1288A+0.0013B+0.2503C_{1}-0.3693C_{2}-0.1815AB-0.1424AC_{1}\\ +0.1462AC_{2}+0.0951BC_{1}+0.1311BC_{2}-0.3955A^{2}-0.1403B^{2} \end{array}$$

(15) $$\begin{array}{l} Y_{5}=5.95+0.1402A+0.0746B+0.0677C_{1}-0.3658C_{2}-0.1890AB-0.1446AC_{1}\\ +0.1848AC_{2}-0.0466BC_{1}+0.2712BC_{2}-0.6180A^{2}-0.2568B^{2} \end{array}$$

where *Y*_1_, *Y*_2_, *Y*_3_, *Y*_4_ and *Y*_5_ are 7d compressive strength, 28d compressive strength, 28d flexural strength, 7d splitting tensile strength and 28d splitting tensile strength, respectively; *A* is the length–diameter ratio of steel fiber; *B* is the content of steel fiber; and *C*_1_ and *C*_2_ are virtual variables of steel fiber types.

The model coefficient shows that the quadratic terms of aspect ratio (*A*) and content (*B*) significantly affect the compressive strength, while fiber type (*C*) mainly dominates the splitting tensile performance through the interaction between linear terms and content.

#### 4.2.3. Prediction Results of the Model

**Figure 25:**
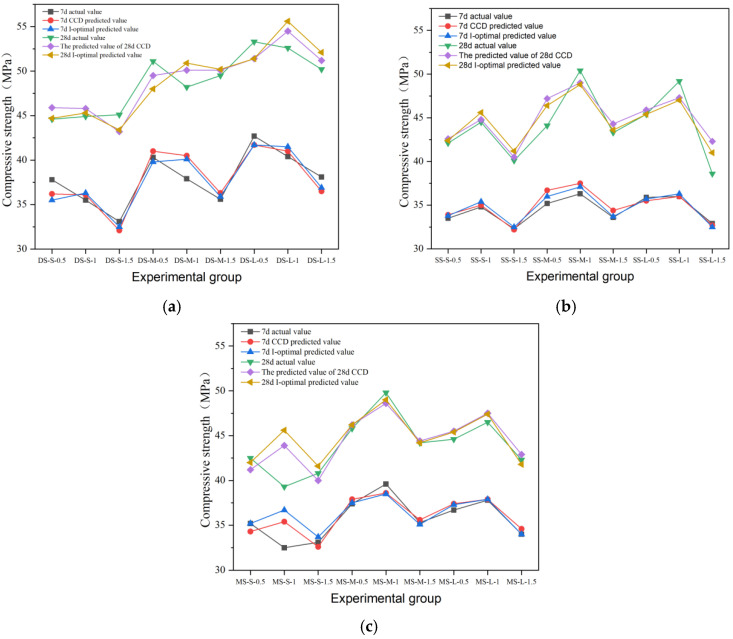
Comparison of the tested and predicted compressive strength of steel-fiber-reinforced concrete: (**a**) end-hook steel fiber; (**b**) shear steel fiber; (**c**) milling steel fiber.

**Figure 26:**
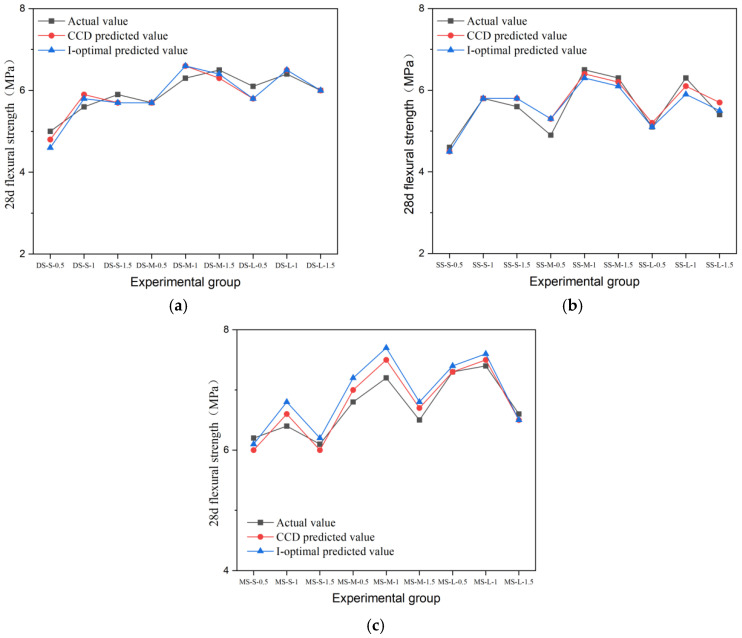
Comparison of the tested and predicted flexural strength of steel-fiber-reinforced concrete: (**a**) end-hook steel fiber; (**b**) shear steel fiber; (**c**) milling steel fiber.

**Figure 27:**
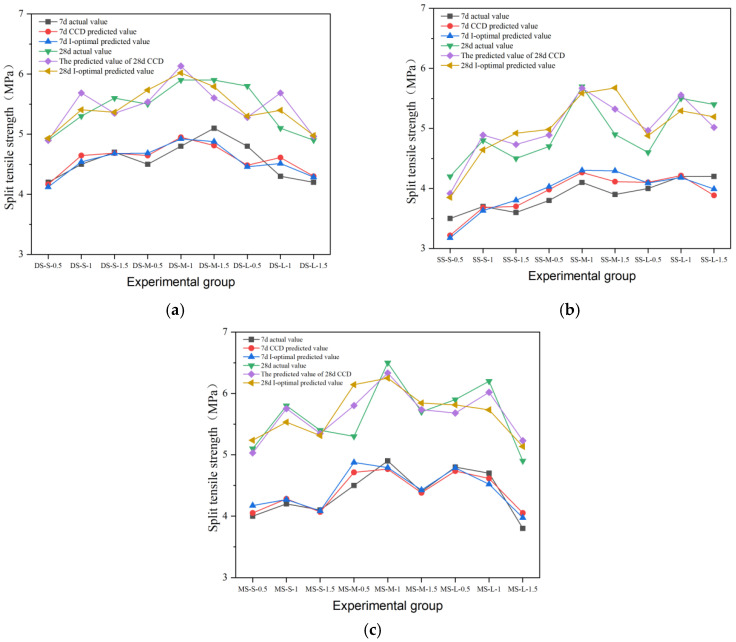
Comparison of the tested and predicted splitting tensile strength of steel-fiber-reinforced concrete: (**a**) end-hook steel fiber; (**b**) shear steel fiber; (**c**) milling steel fiber.

The prediction performance of the I-optimal model was compared with the results of the CCD model. The comparison of predicted versus actual values for compressive, flexural, and splitting tensile strength across different fiber types is shown in Figure 25, Figure 26 and Figure 27. A quantitative comparison of the prediction variance for the two models is presented in Table 17. The data indicate that the I-optimal model achieved lower prediction variance for most response variables, despite using fewer experimental runs, confirming its superior efficiency and prediction stability.

For example, the predicted variance pairs of the two models are shown in Table 17. The data show that the predicted variance of the response variables is basically lower than that of the CCD model, and only the 28d splitting tensile strength model is slightly higher. Among them, the variance of 7-day splitting tensile strength decreased the most. Taking the 28d flexural strength as an example, the prediction variance of the I-optimal model is only 0.063, which means that the 95% confidence interval width of the optimal ratio is 20.3% narrower than that of the CCD model. On the whole, I-optimal response surface design basically achieved a reduction in global prediction variance under the condition that the experimental amount was reduced by 33% and achieved good results.

### 4.3. Determination of Optimal Mix Ratio

#### 4.3.1. Definition of Objective Functions and Constraints

To tackle the challenge of improving several mechanical properties at once, which often work against each other, as shown by the single-objective optima in Table 12, a multi-objective optimization was carried out using the established I-optimal regression models (Equations (11)–(15)). The primary objective of the multi-objective optimization was to maximize the five key mechanical properties of SFRC simultaneously (Y_1_–Y_5_). The optimization was constrained within the experimental design space to ensure the validity of the predictions. The constraints for the independent factors were: 40 ≤ A ≤ 60 (aspect ratio); 0.5% ≤ B ≤ 1.5% (fiber volume content); and C ∈ {End-hook, Shear, Milling} (fiber type). Copper-plated and corroded fibers were excluded from the optimization based on their demonstrated limited reinforcing efficiency.

#### 4.3.2. Desirability Function Approach for Multi-Objective Optimization

Given the conflicting nature of the objectives, a Desirability Function Approach (DFA) was employed to find a single optimal solution that offers the best trade-off among all responses. This method is integrated into the Design-Expert software and is widely used for such problems. In this approach, each predicted response (Y_i_) is first transformed into an individual desirability value (d_i_), which is unitless and ranges from 0 to 1 (d_i_ = 0 represents a completely undesirable response; d_i_ = 1 represents a fully desirable response).

Since the goal for all five mechanical properties is to achieve the highest possible value, the “maximize” desirability function was applied. The individual desirability for each response is calculated as follows (16):

(16) $$d_{i}=\left\{\begin{array}{l} 0\;\;(if\;Y_{i}<Low_{i})\\ \frac{Y_{i}-Low_{i}}{High_{i}-Low_{i}}\;(if\;Low_{i}\leq Y_{i}\leq High_{i})\\ 1\;(if\;Y_{i}>High_{i}) \end{array}\right.$$

where *Low_i_* and *High_i_* are the lower and upper acceptable bounds for the response *i*, typically set to the minimum and maximum predicted values from the model within the design space. Subsequently, these individual desirabilities are combined into a single composite desirability (*D*) by calculating their weighted geometric mean (17):

(17) $$D={\left(\prod_{i=1}^{n}d_{i}^{w_{i}}\right)}^{\frac{1}{\sum_{i=1}^{n}w_{i}}}$$

where *n* is the number of responses (here, *n* = 5) and *w_i_* is the importance weight assigned to each response *i*.

#### 4.3.3. Weighting Strategy and Solution Selection

In this study, as the objective was to achieve a balanced and comprehensive improvement in the overall mechanical properties, equal importance was assigned to all five strength indices. Therefore, the weights were set as *w_i_* = 1 for *i* = 1,2,3,4,5.

The optimization process was then executed by searching the factor space (defined by the constraints on A, B, and C) to find the combination that maximizes the composite desirability D. This ensures that the selected solution is not optimal for just one property but performs well across all five, representing the best compromise. The results of this multi-objective optimization, including the optimal factor settings and the corresponding predicted strengths, are presented in Table 18. The comprehensive optimal ratio identified through I-optimal optimization is highly consistent with the conclusion drawn from the prior CCD analysis. The results indicate that incorporating end-hook fibers with an aspect ratio of 52.43 and a volume content of 0.98% into cement concrete facilitates the collaborative optimization of multiple strength properties. The convergence of the optimal solutions from both design methods confirms the robustness of the underlying relationship between fiber parameters and mechanical response. It also highlights the capability of the I-optimal design to enhance prediction efficiency without compromising the physical interpretability of the models.

## 5. Conclusions

This study systematically investigated the comprehensive effects of steel fiber type, aspect ratio, and volume content on the mechanical properties of cement concrete and compared the performance with copper-plated and corroded fibers. A multi-objective prediction model for the mechanical properties of steel-fiber-reinforced concrete was established using a combined macro- and micro-scale experimental approach and response surface methodology optimization. The main conclusions are as follows:

(1) Steel fibers significantly enhance the overall mechanical properties of concrete. End-hook, shear, and milling steel fibers effectively improve the compressive, flexural, and splitting tensile strength of concrete. Among them, end-hook steel fiber performs best in compressive and splitting tensile strength, increasing the 28-day compressive strength by up to 41%. Milling steel fiber performs best in flexural strength, achieving an improvement of up to 72%. This improvement is primarily attributed to the enhanced bridging effect and frictional bond at the fiber–matrix interface. For milling fibers, their irregular surface texture is considered the primary factor contributing to this enhanced bond, as inferred from their superior flexural performance.
(2) Steel fiber incorporation fundamentally transforms the failure mode of concrete from brittle fracture to quasi-ductile behavior. Observations during the four-point bending tests consistently showed that plain concrete failed suddenly with complete specimen separation at peak load, while all fiber-reinforced specimens maintained structural integrity after first cracking, with fibers bridging the fracture plane and sustaining residual load over larger deformations. This ductility improvement was most pronounced for end-hook and milling fiber types, attributable to their mechanical anchorage and surface interlock mechanisms, respectively. The enhancement of post-crack energy absorption capacity is a critical complement to the strength improvements documented in this study and underscores the structural safety benefits of steel fiber reinforcement.
(3) The reinforcing effect of fibers exhibits a clear synergistic dependency on parameters and an optimal application window. The reinforcing effect is influenced by both the aspect ratio and the volume content, with a clearly identifiable optimal range for each. Compressive strength increased significantly at lower fiber contents (0.5–1.0%), whereas flexural and splitting tensile strength showed a continuing increasing trend with higher content within the tested range. The overall reinforcing effect was optimal when the aspect ratio was within the range of 50–60. Values outside this range weakened the fiber bridging and anchoring effects.
(4) The surface state of the fiber has a decisive influence on its reinforcing effectiveness. The reinforcing effect of copper-plated and corroded steel fibers was significantly lower than that of ordinary steel fibers due to reduced interfacial adhesion. The smooth surface of copper-plated fibers promotes agglomeration, while the oxide layer on corroded fibers weakens the interfacial bond. Both conditions result in limited mechanical improvement, underscoring that the integrity of the fiber–matrix interface is a prerequisite for effective reinforcement.
(5) The response surface methodology (RSM) models, particularly the I-optimal design, demonstrated high-precision predictive capability. The RSM models developed using both CCD and I-optimal designs accurately predicted multiple mechanical properties of fiber-reinforced concrete, exhibiting high goodness-of-fit and low prediction error. The I-optimal method maintained high prediction accuracy even with a 33% reduction in the number of experimental runs compared to the CCD, providing a reliable and efficient tool for the multi-objective optimization of fiber–concrete mix proportions.

This study systematically investigated the influence of key parameters on the mechanical properties of steel-fiber-reinforced concrete and establishes reliable optimization pathways for mixture design. The established prediction models and optimization methodology provide a theoretical basis and technical support for the material design and application of high-performance fiber-reinforced concrete in infrastructure projects such as highways and bridges. Future work should prioritize the quantitative characterization of flexural toughness through systematic recording of full load–deflection curves and calculation of toughness indices in accordance with CECS 13-2009, providing a more rigorous and complete evaluation of the ductility improvements associated with steel fiber incorporation. Additionally, dynamic load response, durability, and long-term performance warrant further investigation.

## Figures and Tables

**Table 1:** Material collection technical indicators.

Value	Water Absorption (%)	Apparent Density (kg/m^3^)	Surface Dry Density (kg/m^3^)	Bulk Density (kg/m^3^)	Crushing Value (%)	Needle Flake Content (%)
Fine aggregate	1.25	2676	2654	2646	-	0.8
Coarse aggregate	0.21	2890	1740	1870	6.4	1

**Table 2:** Overview of various types of fibers.

Material	Type	Surface State	Tensile Strength (MPa)	Length–Diameter Ratio Selection
Steel fiber	End-hook steel fiber	Cold-drawn steel wire	1150	40/50/60
	Shear steel fiber	Cold-rolled strip	450	40/50/60
	Milling steel fiber	Ingot milling pin	650	40/50/60
	Copper-plated steel fiber	Anti-rust treatment	2695	60
	Corroded steel fiber	Rust treatment	-	60

**Table 3:** Technical index of steel fiber.

Inspection Item		Unit	Technical Requirement	Experimental Result
Presentation quality		-	Surface of steel fiber should be clean and dry; no oil stain and other impurities; total weight of bonding sheets, iron pins and impurities contained in steel fibers less than 1%.	Complied with the requirements
Tensile strength	End-hook type	MPa	1000 ≤ fu ≤ 1100	1150
	Shear type	MPa	400 ≤ fu ≤ 700	450
	Milling type	MPa	600 ≤ fu ≤ 1000	650
Bending performance		-	Bending performance of steel fiber: meeting the requirement of less than 95% non-breaking rate in a single 90° bend test.	Non-breaking rate in a single 90° bend test: 100%
Shape qualification rate		%	≥90	100

**Table 4:** Technical index of copper-plated fiber.

Performance Index	Unit	Index Requirements	Test Result
Tensile strength	MPa	≥2085	2695
Length	mm	13 ± 1.3	12.98
Equivalent diameter	mm	0.2 ± 0.02	0.2
Draw ratio	-	65 ± 6.5	65
Bending performance	-	Cold bending 90 20/20 continuously	20/20 continuously

**Table 5:** Cement performance parameter.

Project	Standard Requirement	Test Result
Initial setting time (min)	≥45	159
Final setting time (min)	≤600	270
Fineness (%)	≤10	2.5
3d flexural strength (MPa)	≥3.5	4.5
3d compressive strength (MPa)	≥17.0	21.2
28d flexural strength (MPa)	≥6.5	8.1
28d compressive strength (MPa)	≥42.5	49.0
Loss on ignition (%)	≤5.0	1.93

**Table 6:** Fly ash performance parameters.

Project	Standard Requirement	Test Result
Fineness (%)	≤30	22
Water demand ratio (%)	≤105	97
Water content (%)	≤1	0.4
Loss on ignition (%)	≤8	2.7

**Table 7:** Slag powder performance parameters.

Project	Standard Requirements	Measured Results
Density (g/cm^3^)	≥2.8	2.91
Specific surface area/(m^2^/kg)	≥400	424
7d total activity (%)	≥70	82
28d total activity (%)	≥95	110
Mobility ratio (%)	≥95	103
Water content (%)	≤1.0	0.2
Loss on ignition (%)	≤3.0	1.1

**Table 8:** Benchmark mix ratio of steel-fiber-reinforced concrete.

Component	Cement	Water	Sand	Stone	Slag Powder	Fly Ash	Additive
Proportion	1	0.566	3.41	3.98	0.305	0.261	0.003

**Table 9:** Aggregate grading.

Mesh Size (mm)	Mass Percentage Passing Through Sieve Holes with Different Apertures (%)											
	26.5	19	16	13.2	9.5	4.75	2.36	1.18	0.6	0.3	0.15	0.075
Value of Pass Rate (%)	100	95	95	95	50	46	27	18	12	6	3	2

**Table 10:** CCD experimental design matrix and results for mechanical properties.

Experimental Group	A	B (%)	C	7d Compressive Strength (MPa)	28d Compressive Strength (MPa)	28d Flexural Strength (MPa)	7d Splitting Tensile Strength (MPa)	28d Splitting Tensile Strength (MPa)
1	50	0.5	End hook	40.3	49	5.7	4.5	5.5
2	60	0.5	Shear	35.9	45.4	5.1	4	4.6
3	60	1.5	Milling	34	42.3	6.6	3.8	5
4	40	0.5	Shear	33.5	42.1	4.6	3.5	4.2
5	50	1.5	Milling	35.3	44.2	6.5	4.4	5.7
6	50	1	Milling	41.3	52.7	7.4	4.8	6.6
7	50	1	End hook	41.2	52.1	6.7	5.1	6.2
8	40	0.5	End hook	37.8	44.6	5	4.2	4.9
9	60	1	Shear	36	49.2	6.3	4.2	5.5
10	60	0.5	Milling	36.7	44.6	7.3	4.8	5.9
11	60	1	End hook	40.4	53.6	6.4	4.3	5.1
12	50	1	Shear	39.5	52.2	6.8	4.5	5.9
13	40	0.5	Milling	35.2	42.5	6.2	4	5.1
14	40	1.5	Shear	32.3	40.1	5.6	3.6	4.5
15	60	1	Milling	37.8	46.5	7.4	4.7	6.2
16	40	1.5	End hook	33.1	45.1	5.9	4.7	5.6
17	50	1.5	End hook	35.6	49.5	6.5	5.1	5.9
18	40	1	Milling	32.5	39.3	6.4	4.2	5.8
19	50	1.5	Shear	33.6	43.3	6.3	3.9	4.9
20	50	1	Shear	36.3	50.4	6.5	4.1	5.7
21	40	1	End hook	35.5	44.9	5.6	4.5	5.3
22	50	1	End hook	37.9	49.2	6.3	4.8	5.9
23	50	1	Shear	40.2	51	6.4	4.2	6.1
24	40	1.5	Milling	33.1	40.8	6.1	4.1	5.4
25	60	1.5	End hook	38.1	53.9	6	4.2	4.9
26	40	1	Shear	34.8	44.5	5.8	3.7	4.8
27	50	1	End hook	39.9	51.6	6.4	5	6.3
28	60	0.5	End hook	42.7	53.3	6.1	4.8	5.8
29	50	0.5	Shear	35.2	44.1	4.9	3.8	4.7
30	50	1	Milling	38.6	48.8	7.7	5	6.1
31	50	0.5	Milling	37.4	45.8	6.8	4.5	5.3
32	50	1	Milling	39.6	49.8	7.6	4.9	6.5
33	60	1.5	Shear	31.8	39.6	5.4	4.2	5.4

**Table 11:** Analysis of variance for the CCD experimental models.

Strength Index		7d Compressive Strength (MPa)	28d Compressive Strength (MPa)	28d Flexural Strength (MPa)	7d Splitting Tensile Strength (MPa)	28d Splitting Tensile Strength (MPa)
*p*-value	Model	<0.0001	<0.0001	<0.0001	<0.0001	<0.0001
	A	0.0010	0.003	<0.0001	0.0115	0.0663
	B	0.0004	0.2367	0.0068	0.9128	0.3787
	C	0.0007	0.0004	<0.0001	<0.0001	0.0005
	AB	0.5391	0.6159	0.0152	0.0096	0.0648
	AC	0.1206	0.0840	0.1335	0.0878	0.2561
	BC	0.3409	0.4754	0.0008	0.0988	0.4351
Misfitting term		0.6909	0.0913	0.1998	0.1876	0.1069
Predicted R^2^		0.7183	0.6941	0.9143	0.7749	0.8010
Adjusted R^2^		0.8151	0.7993	0.9598	0.8523	0.9067

**Table 12:** Classification of optimal influencing factors.

Strength Type	Optimal Length-Diameter Ratio	Optimal Dosage (%)	Optimal Fiber Type	Strength Value (MPa)
7d compressive strength	56.668	0.672	End-hook type	42.131
28d compressive strength	59.228	1.155	End-hook type	54.294
28d flexural strength	55.780	0.880	Milling type	7.617
7d splitting tensile strength	49.124	1.112	End-hook type	4.959
28d splitting tensile strength	51.613	0.972	Milling type	6.345

**Table 13:** Comprehensive optimal factor combination for multi-objective optimization.

Comprehensive Optimal Conditions of CCD Experiment	
Draw ratio	52.497
Volume content (%)	0.966
Fiber type	End-hook type
Strength type	Optimal strength value (MPa)
7d compressive strength	41.189
28d compressive strength	53.962
28d flexural strength	6.655
7d splitting tensile strength	4.922
28d splitting tensile strength	6.104

**Table 14:** The difference between the two optimization design methods.

Trait	I-Optimal	D-Optimal
Optimization objective	Minimize the average variance of the predicted response	Maximize the accuracy of parameter estimation
Mathematical form	$\min\int \mathrm{Var}\left[\hat{y}(x)\right]dx$	$Max\det(X^{T}X)$
Applicable scenario	Parameter optimization (finding the best combination)	Factor significance analysis
Output focus	Flat area of the response surface	Confidence interval of model coefficients

**Table 15:** Experimental design and results based on the I-optimal method.

Experimental Group	A	B (%)	C	7d Compressive Strength (MPa)	28d Compressive Strength (MPa)	28d Compressive Strength (MPa)	7d Splitting Tensile Strength (MPa)	28d Splitting Tensile Strength (MPa)
1	50	0.5	End hook	40.3	49	5.7	4.5	5.5
2	60	1	End hook	40.4	53.6	6.4	4.3	5.1
3	60	1.5	End hook	38.1	53.9	6	4.2	4.9
4	40	1	End hook	35.5	44.9	5.6	4.5	5.3
5	40	1.5	End hook	33.1	45.1	5.9	4.7	5.6
6	60	0.5	End hook	42.7	53.3	6.1	4.8	5.8
7	50	1.5	End hook	35.6	49.5	6.5	5.1	5.9
8	50	1	End hook	37.9	49.2	6.3	4.8	5.9
9	40	1	End hook	37.5	45.8	5.8	4.6	5.4
10	60	0.5	Shear	35.9	45.4	5.1	4	4.6
11	40	1.5	Shear	32.3	40.1	5.6	3.6	4.5
12	50	1	Shear	36.3	50.4	6.5	4.1	5.7
13	40	0.5	Shear	33.5	42.1	4.6	3.5	4.2
14	50	0.5	Shear	35.2	44.1	4.9	3.8	4.7
15	60	1.5	Shear	31.8	39.6	5.4	4.2	5.4
16	50	1	Shear	39.5	52.2	6.8	4.5	5.9
17	50	1	Mill	39.6	49.8	7.6	4.9	6.5
18	40	0.5	Mill	35.2	42.5	6.2	4	5.1
19	40	1.5	Mill	33.1	40.8	6.1	4.1	5.4
20	60	0.5	Mill	36.7	44.6	7.3	4.8	5.9
21	60	1.5	Mill	34	42.3	6.6	3.8	5
22	50	1	Mill	38.6	48.8	7.7	5	6.1

**Table 16:** I-optimal optimization model parameters.

Model (Quadratic)	p_1_	p_2_ (Missing Item)	Sum of Squares	Freedom	Mean Square	Adjusted R^2^	Predicted R^2^
7d compressive strength	0.0025	0.6576	159.25	11	14.48	0.8830	0.7542
28d compressive strength	0.0037	0.0506	372.34	11	33.85	0.8725	0.7323
28d flexural strength	0.0001	0.1249	12.63	11	1.15	0.9410	0.8762
7d splitting tensile strength	0.0105	0.1978	3.73	11	0.3394	0.8382	0.7602
28d splitting tensile strength	0.0156	0.1178	5.73	11	0.5213	0.9223	0.7969

**Table 17:** Comparison of the prediction variance of the two models.

Model	CCD Model Predicted Variance	I-Optimal Model Predicted Variance
7d compressive strength	2.471	2.111
28d compressive strength	5.944	5.443
28d flexural strength	0.079	0.063
7d splitting tensile strength	0.072	0.045
28d splitting tensile strength	0.116	0.124

**Table 18:** Comprehensive optimal influencing factors after I-optimal optimization design.

Comprehensive Optimal Condition	
Draw ratio	52.431
Volume content (%)	0.978
Fiber type	End-hook type
Strength type	Optimal strength value (MPa)
7d compressive strength	40.778
28d compressive strength	53.971
28d flexural strength	6.699
7d splitting tensile strength	4.893
28d splitting tensile strength	5.982

## Data Availability

The original contributions presented in this study are included in the article. Further inquiries can be directed to the corresponding author.
